# Oncology Pharmacists Can Reduce the Projected Shortfall in Cancer Patient Visits: Projections for Years 2020 to 2025

**DOI:** 10.3390/pharmacy8010043

**Published:** 2020-03-18

**Authors:** Katherine Knapp, Robert Ignoffo

**Affiliations:** Touro University California College of Pharmacy, Vallejo, CA 94592, USA; Robert.ignoffo@tu.edu

**Keywords:** oncology pharmacists, Board Certified oncology pharmacists, oncology pharmacy residencies, oncology pharmacy residents, nurse practitioners, physician assistants, cancer care models, advanced practice providers, cancer trends, cancer incidence, cancer mortality, cancer survivorship, and cancer statistics

## Abstract

Based on the projected need for a larger oncology care workforce, we estimated the patient care visits and care activities that Board Certified oncology pharmacists (BCOPs) could contribute to oncology care from 2020–2025. Using projected counts for BCOPs through 2025, we estimated that 2.9–4.1 million 30-min BCOP patient visits were possible at 50% workforce capacity. BCOPs’ clinical activities overlapped strongly with those of nurse practitioners (NPs) and physician assistants (PAs) in patient education and treatment management. BCOPs could help reduce provider stress and burnout concerns by spreading these activities across a broader set of providers. BCOPs were more active than NPs and PAs in clinical trials research. Recent advances in immunotherapy, pharmacogenetics, pharmacogenomics, and oral oncolytic agents make the medication-focused training of OPs particularly useful to care teams. Comparison also showed that BCOPs were less active in providing follow-up visits and prescribing. Fulfilling the projected BCOP numbers through 2025 will require continued growth in Postgraduate Year 2 (PGY2) oncology pharmacy resident programs and on-the-job training programs. Our review of the trends in cancer incidence, mortality, and survivorship suggest a sustained need for the activities of BCOPs and other oncology care providers.

## 1. Introduction

In 2016 we published an article entitled “Role of the Oncology Pharmacist in Reducing the Shortfall in Oncology Patient Visits” [1] in response to the work of Eriksen and colleagues in 2007 [2,3], who warned that the supply of oncologists would not be able to meet the demand for oncology services by 2020. We showed that oncology-trained pharmacists could have a positive impact on increasing the number patient visits. This paper is an update of our previous report on the number of oncology pharmacists (OPs) in reducing patient visits and also describes their clinical activities as they relate to other providers of oncology care.

According to a 2014 American Society of Clinical Oncology (ASCO) report, the demand for oncology treatment is expected to increase by 40% by 2025 [4]. However, it was predicted that there would be a shortage of 2200 oncology physicians by the year 2025. Compounding this problem, in a 2019 survey, in 37 of the 50 metropolitan areas studied, at least 20% of practicing oncologists were over the age of 65 [5]. Based on this demographic, it was suggested that the retirement of older physicians could limit patients’ access to oncology care. In addition, other factors such as the increasing number of new drugs and those being studied in research clinical trials, the increasing caseload and complexity of care, along with inefficient patient care would contribute to an increased demand for oncology services [6]. Possibly countering these trends, recent reports indicate a reduced cancer incidence [7], a reduced mortality from cancer [8,9] and an increase in the number of cancer survivors [10,11], which could either increase or decrease the demand for oncology services.

The earlier-cited studies by ASCO and a report by Erikson et al. in 2007 estimated that, under the scenario of high supply and low demand, the projected shortfall in patient visits would be 9.4 million by 2020. To stem the impact of the projected oncology physician shortage, oncology practices have utilized the services of Advanced Practice Providers (APPs), namely nurse practitioners (NPs) and physician assistants (PAs). In 2007, it was estimated that NP/PAs could increase patient visits by 0.8 to 3.4 million by 2020 [2,3]. A more recent 2018 study updated the progress in incorporating NPs and PAs in oncology care teams noting that despite growth in both Advanced Practice Provider (APP) groups, a shortfall in projected patient visits persists [12]. This study identified 5350 APPs who were involved in cancer care and considered to be vital in ensuring access to quality patient care.

Based on the reports of a continuing shortfall in visits, a 2016 study addressed the question of whether OPs could also contribute to increasing available patient visits [1]. The study estimated there would be 3639 OPs with training and/or experience in working clinically with oncology patients by 2020. The study used a Delphi process to identify eight practice-related activities that ≥ 80% of OPs perform on a routine basis. The study also estimated the contribution in the form of 30-min oncology patient visits that these pharmacists could make. It was estimated that, at 50% work capacity, OPs could provide 2.6–3.3 million 30-min visits by 2020 and suggested that these pharmacists could help address the shortfall in visits [1]. Furthermore, the contributions and value of OPs were extensively described in a 2019 study by Segal et al. which reviewed 66 peer-reviewed studies demonstrating the value and activities of the oncology pharmacist [13]. To complement these efforts and their implications for including pharmacists more formally into the oncology care team, we have updated the numbers of both oncology pharmacy residents and Board Certified OPs (BCOPs) projecting from year 2020 through to the year 2025.

Several of the studies cited here utilize data from the 2007 reports from Erikson et al. and ASCO [2,3]. The data cited in the 2007 reports—particularly those used to estimate the numbers of needed patient visits—date back to 2005. To the best of our knowledge, these data have not been updated, so while the focus of this study is on oncology pharmacist contributions to patient care, there is a need also to consider, at least briefly, whether the changes in incidence, mortality, and survivorship could affect the demand for oncology pharmacy services.

The purposes of this study were: (1) to project the number of BCOPs from 2020 through 2025; (2) to update the potential contribution of OPs in providing services to patients in 30-min visits; (3) to compare the routine clinical activities of OPs with those of NPs and PAs in providing clinical services; and (4) to review those changes in cancer statistics that could affect the demand for OPs.

## 2. Materials and Methods

Data regarding PGY2 pharmacy oncology residents were drawn from an earlier study from 2008 to 2013 [1] and supplemented by ASHP data for 2014 to 2019 [14]. Data regarding United States (US) BCOPs from 2008 to 2019 were provided by the Board of Pharmacist Specialties (personal communication, BPS staff). The data only included US-based BCOPs, a difference from an earlier study which included BCOPs not based in the US [1]. The data were plotted using Excel (Microsoft Excel Office 365, Redmond, WA). Trend lines for each variable were added using Excel and choosing the trend equation with the highest R^2^ for making projections. The trend equations with the best fit were used to calculate the projected values for 2020 through 2025. 

We estimated the number of 30-min oncology patient visits that could be provided by available BCOPs using the methods described in an earlier study [1]. Patient visits were calculated using the following formula: Total patient visits = number of BCOPs by year × 41 patient visits (an average of 41 patients per week was obtained from our previous study [1]) by BCOPs per week × 48 weeks per year × 0.85 (0.85 is a correction factor taken from the Association of American Medical Colleges (AAMC) study that relates to other activities that reduce time for patient care, such as drug distribution, administrative duties, and teaching [3]).

We compared the routine activities of OPs [1] to those of NPs and PAs [12]. From the activity lists in the two studies, we only compared those activities performed by ≥ 80% of either group. For treatment-related activities, the oncology pharmacist list had five activities—adjusting chemotherapy, assessing chemotherapy response and related toxicities, managing nausea and vomiting antiemetic therapy, managing other symptoms, providing supportive care, and managing pain—that were all judged to fall within the single PA/NP activity termed “treatment management”. The five pharmacist activities were combined, and their percentages averaged to make the comparison. 

Finally, we reviewed cancer statistics for the trends in incidence, mortality, and survival rates and related them to care activities that would likely involve OPs. We searched PubMed, Google Scholar (google.com), the websites of the National Cancer Institute (www.cancer.gov), the American Cancer Society (www.cancer.org), and the American Society of Clinical Oncology (www.asco.org) using these keywords: cancer trends, cancer incidence, cancer mortality, cancer survivorship, and cancer statistics. The resulting literature was reviewed focusing on the possible impact of these cancer trends on the oncology pharmacy workforce.

## 3. Results

**Figure 1:**
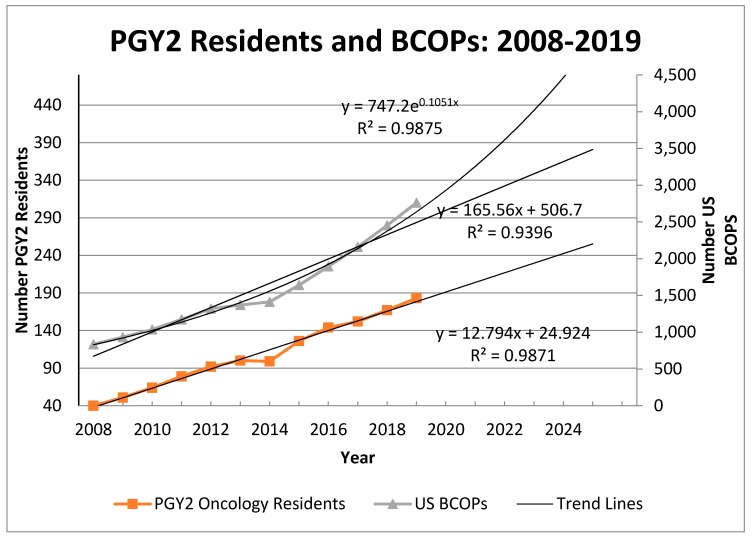
Growth of PGY-2 Residents and US-Based BCOPs: 2008–2019.

Figure 1 shows the numbers of PGY2 oncology residents and BCOPs from 2008 to 2020 with trend lines for each variable. The trend equation for residents showed a good fit using a linear trend model (R^2^ = 0.99). BCOPs increased in number from 836 in 2008 to 2761 in 2019. For the BCOP data, an exponential trending equation (R^2^ = 0.99) provided a slightly better fit than a linear model (R^2^ = 0.94). The reported values for US BCOPs were consistently lower than those reported in an earlier study because that study included BCOPs located outside the US [1]. For PGY2 oncology residents, the projections are 178 in 2020 and 242 in 2025. For BCOP numbers, the 2020 projections are 2532 (linear trend) and 2930 (exponential trend); and the 2025 projections are 2980 (linear trend) and 4955 (exponential trend). We noted that the 2020 linear projection for BCOPs (n = 2532) is less than the 2019 count (n = 2761) due to the 2019 value falling above the trend line.

Using the projection data, Table 1 shows the range of 30-min patient visits that could be provided by OPs from 2020 through to 2025. Our results show that the number of potential 30-min patient visits provided by OPs at 50% work capacity is 2.2 to 2.4 million (M) in 2020, which is less than our previous estimate of 2.5 to 3.5 M [1]. In 2025, the estimates are 5.8M (linear model) to 8.3M (exponential model). This reflects a 31%–41% increase in the number of visits for the linear and exponential models, respectively. The increase is due to the upturn in the number of oncology-trained pharmacists.

**Figure 2:**
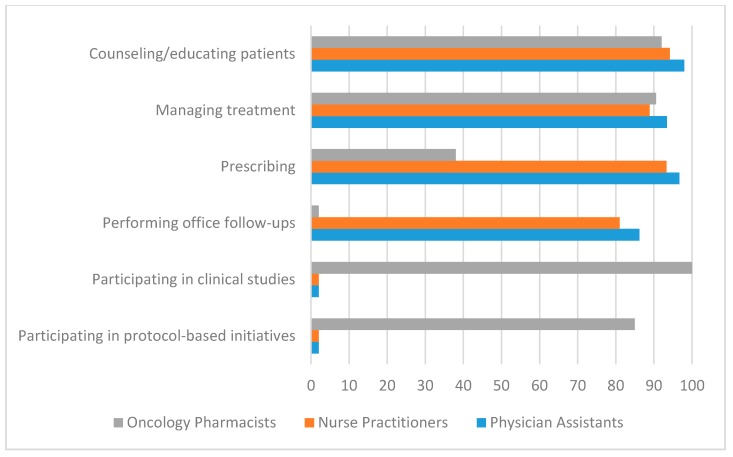
Comparison of Oncology Pharmacist and NP/PA activities.

Figure 2 compares the predominant clinical activities of OPs to those reported for NPs and PAs [1,12]. Only those activities reported at ≥80% are shown with one exception in the prescribing category. While the percentages reported reflect slightly different measures, the studies reported those activities that occur “frequently or routinely” (for OPs) or “percent who report this care type” (for NPs and PAs). The comparison shows NPs, PAs, and OPs heavily and routinely involved in “educating/counseling patients” and “managing treatment”. Substantial differences are noted for the other service/activity areas. For NPs and PAs, “prescribing” is listed as a routine activity (NPs 93%, PAs 97%) while OPs identify “ordering routine chemotherapy” at 38%. Involvement in clinical studies is cited at 100% for OPs while “research” is cited separately from “direct care” for NPs and PAs with a median reported value close to 0%. Also, NPs and PAs report “performing follow-up office visits” at 81% (NPs) and 86% (PAs), while Figure 2 suggests that OPs are not routinely involved in performing follow-up visits. We noted, however, that office-based follow-up visits were not addressed in the OP study and hence the comparisons may not be valid [1].

The cancer incidence and mortality rates for all cancers in the US currently show downward trends [6,7,8,9,10,15]. The trends largely reflect earlier detection, new therapies, prevention, and lifestyle changes. These trends relate to decreases in prostate cancer (early detection), melanoma (new therapies), lung cancer (new therapies and lifestyle changes), and cervical cancer (prevention) [9]. The cancer incidence rates are reported in terms of new diagnoses per 100,000 population. So while the incidence of cancers is trending downward, the combination of US population growth and decreased mortality work together to increase the number of people considered “cancer patients” over time. Survivorship statistics reflect these relationships and show an upward trend triggering what has been described as a challenge to the capacity of the oncology workforce [6].

OPs are generally not involved in the diagnosis of cancers but, as shown in Figure 2, are heavily involved in treatment management. The demand for the treatment-related activities cited earlier—adjusting chemotherapy, assessing chemotherapy response and related toxicities, managing nausea and vomiting antiemetic therapy, managing other symptoms, providing supportive care, and managing pain—is likely to increase proportionately to patient numbers. Clinical studies involving new drugs and as-yet undetermined complications of new therapies are likely to increase the utilization of OPs, whose training focuses on medications and commonly involves working in clinical studies. As survivorship increases, the demand for follow-up care for long-term complications, monitoring, and patient education may include OPs operating in clinic-type facilities such as those that are now common for diabetes and blood pressure management [5]. Overall, the current cancer statistics suggest a sustained demand for OPs and their specialty skills. 

## 4. Discussion

This study shows that PGY2 oncology residents continue to grow annually and in a linear fashion, and there are currently 183 residents for the year 2019 (Figure 1). The BCOP growth rate has accelerated in recent years, providing some confidence that the projections for providing patient visits are achievable at least at the linear growth level. To further confirm achievability, the formula for projecting patient visits uses an 85% correction factor to allow for other activities outside of patient visits. It cannot be assumed, however, that the accelerated growth rate will continue and growth trends should be monitored. In addition, we note that the number of BCOPs grew by 307 from 2018 to 2019. This growth exceeds the number of PGY2 oncology residents over the same period by 291 (data not shown). The rather large difference suggests that more oncology pharmacists are qualifying for BCOP status through on-the-job training rather than completing a PGY2 oncology residency. To maintain the recent BCOP growth, we encourage oncology programs to continue expanding the number of PGY2 oncology residents, to provide on-the-job training for pharmacists who want to specialize in oncology pharmacy, and to support their pharmacists to achieve BCOP status.

In late 2019, the National Academy of Sciences held a workshop entitled “Developing and Sustaining an Effective and Resilient Oncology Careforce” [16]. Several participants described the different factors that could impact cancer care, including the capacity of the careforce. One of the speakers mentioned that one way to extend the capacity of the cancer care team would be to have oncology pharmacists (OPs) involved in patient navigation, treatment, and palliative care. These notions were supported by a nurse from the Office of Nursing Affairs of the Veterans Affairs, stating that OPs were already extending capacity by managing oral oncology clinics. Another participant stated that incorporating pharmacists into the palliative care team was a cost-effective strategy for increased patient services [17].

With the growing number of cancer survivors along with the physician shortage, the ability of oncologists to care for patients has become a great burden. In addition, new payment models, more interprofessional collaborative practice, and the widespread adoption of new technologies have added complexity to the health care system, which can lead to stress and burnout [11]. As a result, oncologists will need to be increasingly more efficient in order to provide quality care while seeing more patients during a workday. OPs can help oncologists and NPs/PAs by freeing up more time for them to perform diagnoses and procedures on their patients. OPs are an optimal health professional to counsel patients about their medication treatment plan. In addition, OPs would enhance efficiency by providing expertise on investigational new drugs and the many new oral oncology drugs, immunologic agents, and supportive care medications that are being used in practice. OPs have the ability to oversee the administration of oral oncology drugs because of their extensive knowledge of drug administration, monitoring of therapy, toxicity management, adherence assessments, and issues concerning pre-authorizations and reimbursement [18]. While data about OP involvement in follow-up visits were not available, it is likely that OPs can provide the services listed above to cancer survivors and those undergoing recurrent medication therapy. OP success will depend on how well they both coordinate with other healthcare providers and communicate/follow-up with patients. Other areas adding to the complexity include pharmacogenomics and pharmacogenetics, which are being incorporated as a therapeutic strategy more frequently in the clinic setting and pose another challenge to the healthcare team. OPs at several universities have taken the lead in the oncology clinic, being involved in molecular tumor boards, and providing consultations on personalized medicine, which is an emerging pharmacy practice role [19].

With regard to the clinical activities of OPs compared to NPs/PAs, there are several which overlap and some that are distinctly different. There is an overlap in patient counseling and treatment management, the latter of which consists of OPs adjusting chemotherapy doses, assessing response and toxicity, managing chemotherapy-induced nausea and vomiting, other symptom management, and pain management. Overlap in activities could be beneficial in that OPs might perform these services when NPs/PAs are involved in other clinical activities. In general, the prescribing activities for pharmacists are limited by state-based scope of practice laws; however, a recent study cites the benefits of expanded OP prescribing in other countries [1,13]. The amount of training and education that OPs receive in the therapeutics of cancer drugs is extensive, which makes them a valuable member of the team that can address issues that patients may have during the course of their therapy. Thus, deploying OPs in many of these endeavors could be an effective way to extend quality oncology care. 

Vulag and colleagues looked at the impact of OPs on Quality Oncology Practice Initiative (QOPI) quality metrics [20]. Of 177 metrics, it was determined that OPs have a positive impact on 66 QOPI measures, which primarily involved optimizing drug therapy, patient counseling, and symptom management. These measures can affect reimbursement; for example, several of the QOPI interventions related to the Centers for Medicare and Medicaid Services Merit-Based Incentive Payment System.

## 5. Limitations

The projected counts for PGY2 residents are based on data from 2008 through 2019. Resident funding, changes in healthcare reimbursement, or other factors could either increase or decrease the number of trained oncology residents and/or the number of pharmacists pursuing BCOP status in the future. Likewise, the projections for patient visits are based on 2005 and earlier data that have not been updated. New therapies and cancer incidence, mortality, and survivorship changes could either increase or decrease patient visit projections. The activities of NPs, PAs, and OPs are governed by state-based scope of practice laws, which can change, thus affecting the activities reported here. The comparison of clinical activities came from two separate studies and required subjective analysis by the authors. The development and approval of new cancer medications, which are occurring at a rapid rate, could affect the demand for oncology services, practice patterns, and cancer statistics beyond what is envisioned in this study. More research is needed to determine if the trends in cancer statistics will further impact the utilization of OPs.

## 6. Conclusions

Assuming a continuing shortfall in the demand for oncology patient care in terms of 30-min visits, we have made a case that OPs can help reduce this shortfall. Projections based on 12 years of data indicate continually increasing numbers of BCOPs and between 2.9–4.1 M additional 30-min visits at 50% BCOP work capacity. BCOP growth is supported heavily by continued PGY2 oncology resident growth and by on-the-job training. The range for projected visits by OPs depends on the growth rate of BCOPs, which has recently been trending upward toward a logarithmic model. The overlap between the clinical activities of OPs and NPs/PAs suggests that more OP involvement could reduce burnout by allowing some clinical activities to be spread across a broader set of providers. Recent advances in immunotherapy, pharmacogenetics, pharmacogenomics, and oral oncolytic agents make the medication-focused training of OPs particularly useful to care teams. Overall, this study suggests that the improved efficiency and effectiveness of oncology care, recognized as a national healthcare priority, would benefit by more inclusion of OPs on oncology care teams.

## Figures and Tables

**Table 1:** Estimated 30-Minute Patient Visits by OPs Annually: Projections 2020 to 2025. Using Exponential and Linear Models *.

Year	Number of BCOPs: Exponential Growth Trend	Exponential Trend: 30-Minute Visits at 50% Availability	Number of BCOPs: Linear Growth Trend	Linear Trend: 30-Minute Visits at 50% Availability
2020	2930	2,450,652	2659	2,223,988
2021	3254	2,721,646	2825	2,362,830
2022	3615	3,023,586	2990	2,500,836
2023	4016	3,358,982	3156	2,639,678
2024	4461	3,731,180	3321	2,777,684
2025	4955	4,144,362	3487	2,916,527

* Based on an assumption that OPs average 41 30-min patient visits per week based on a previous study [1].
